# Immunohistochemical Reactivity of the 14F7 Monoclonal Antibody Raised against N-Glycolyl GM3 Ganglioside in Some Benign and Malignant Skin Neoplasms

**DOI:** 10.5402/2011/848909

**Published:** 2011-04-10

**Authors:** Rancés Blanco, Enrique Rengifo, Charles E. Rengifo, Mercedes Cedeño, Milagros Frómeta, Adriana Carr

**Affiliations:** ^1^Department of Quality Control, Center of Molecular Immunology, Havana 11600, Cuba; ^2^Department of Pathology, Manuel Fajardo General Hospital, Havana 10400, Cuba; ^3^Research and Development Direction, Center of Molecular Immunology, 216 Street, 15 Avenue, Atabey, Playa, P.O. Box 16040, Havana 11600, Cuba

## Abstract

The evaluation of 14F7 Mab (anti-N-glycolyl GM3 ganglioside) immunorecognition in normal skin, cutaneous malignant melanoma (CMM), and in lymph node metastases (LNM) has been previously reported. In this work we extended the study to benign (BMN) and dysplastic (DMN) melanocytic nevi, basal (BCC), and squamous cell carcinoma (SCC). Immunohistochemical assays with 14F7 followed by a biotinylated link universal and streptavidin-AP in normal and pathological tissues were made. No reaction of 14F7 in normal skin (0/10) as well as a low reactivity in BMN (2/11) and DMN (1/7) was detected. A limited staining in BCC (2/13) and in SCC (4/8) was also evidenced, while 14F7 Mab were mostly reactive in CMM (28/28) and in LNM (6/7). These results suggest that 14F7 reactivity could be closely related with the more aggressive biological behavior of CMM and also support the use of NeuGcGM3 as target for both passive and active melanoma immunotherapy.

## 1. Introduction

The incidence of human skin cancer has significantly increased in the last decades [1]. While basal cell carcinoma (BCC) and squamous cell carcinoma (SCC) are malignant neoplasms with a low lethality and a good prognosis [2], the 10-year survival rate is 92% among the patients with T1 melanomas, and is 50% in patients with T4 melanomas [3].

Gangliosides are glycosphingolipids that contain one or more sialic acid residues and are involved in a wide variety of biological events that occur in the cell membranes of vertebrates. These molecules have an important role in tumoral progression, as well as in metastatic potential of some malignant neoplasms [4, 5]. The events that take place during the malignant transformation of the cells, which lead to the establishment of qualitative and quantitative alterations in the gangliosides composition, have been extensively documented [6–8]. These changes allow considering some gangliosides as tumor-associated antigens and they have been selected as possible target for active and passive immunotherapy [9, 10]. The aberrant expression of N-glycosylated gangliosides has been identified in several malignancies using immunohistochemical methods [10, 11]. 

The N-glycolyl GM3 ganglioside (NeuGcGM3) expression in cutaneous melanoma and breast ductal carcinoma using 14F7 Mab, as well as its limited presence in normal adult human tissues was previously reported by our group [10]. More recently, the study was extended to cutaneous melanomas and their metastases [12] and to epithelial tumors of the digestive system [13]. Additionally, the expression of NeuGcGM3 in nonsmall cell lung cancer as well as in Wilms tumor has been also reported [14, 15]. 

The analysis of 14F7 Mab immunoreactivity could be useful in order to extend the assessment of this molecule as target for cancer immunotherapy as well as to look for a better understanding of the differences in the biological behavior of the primary malignancies of the human skin. Here we show the evaluation of the 14F7 Mab recognition in other benign and malignant entities of human skin such as benign (BMN) and dysplastic melanocytic nevi (DMN), BCC, and SCC. CMM, lymph node metastases and samples of normal skin were also included in the study.

## 2. Materials and Methods

### 2.1. Monoclonal Antibody

We used 14F7 Mab, produced at the Center of Molecular Immunology (Havana, Cuba) as previously described [10].

### 2.2. Tissue Specimens

Ten normal skin samples, 11 benign melanocytic nevi, 7 dysplastic nevi, 8 squamous cell carcinomas, 13 basal cell carcinomas, 28 cutaneous melanomas, and 7 lymph node metastases formalin-fixed and paraffin embedded tissues from Manuel Fajardo General Hospital and the National Institute of Oncology and Radiobiology were obtained, after approved consent by the institutional ethical committees.

### 2.3. Immunohistochemical Procedure

Five micron sections of formalin-fixed and paraffin-embedded tissues were obtained in a microtome (Leitz 1512). The samples were kept at 70°C for 1 h, dewaxed and rehydrated in a descending ethanol series and kept in distilled water for 10 minutes and then TBS solution for 5 minutes. Afterward the reactivity of total tissue proteins was blocked with a commercial solution (Dako X0909, Carpinteria, USA) for 10 minutes as described [13]. The slides were incubated with 14F7 Mab (10 *μ*g/mL) for 1 h in humid chamber, then incubated with a biotinylated link universal and streptavidin-AP (Dako K0678, Carpinteria, USA) for 30 minutes each step. Replacement of 14F7 by TBS and a sample of breast infiltrating ductal carcinoma [10] were used as negative and positive controls, respectively. Between incubations the samples were washed with TBS for 10 minutes. The enzyme activity was detected with a commercial solution of fuchsin (a red chromogen) (Dako K0678, Carpinteria, USA) in order to avoid confusion with melanin. Afterward, the tissues were counterstained with Mayer's Hematoxylin (Dako S2020, Carpinteria, USA) and the slides were mounted with aqueous mounting medium (Dako S3025, Carpinteria, USA).

### 2.4. Evaluation of Results

The intensity of the reaction was qualitatively estimated and expressed as follows: negative (−), weak (+), moderate (++), and intense (+++) and combinations of these patterns were used to express intermediate levels of expression. The percentages of immunoreactive cells (positive cells) were estimated in the most representative areas of tumors with a 10X lens and scored from 0 to 3, where 0 represents the absent of immunostaining (negative up to 5%), 1 (6–25%), 2 (26–50 %) and 3 (more than 50%) of the cells exhibiting staining. The results of two observers were considered as the final evaluation.

## 3. Results

### 3.1. Immunohistochemical Staining in Normal and Nonmalignant Lesions of Human Skin

The results of 14F7 Mab immunoreactivity in some normal and nonmalignant formalin-fixed and paraffin-embedded human skin samples are showed in Table 1.

### 3.2. Normal Skin

No immunorecognition of 14F7 Mab in normal melanocytes and keratinocytes (0/10) was observed.

### 3.3. Benign and Dysplastic Melanocytic Nevi

**Figure 1:**
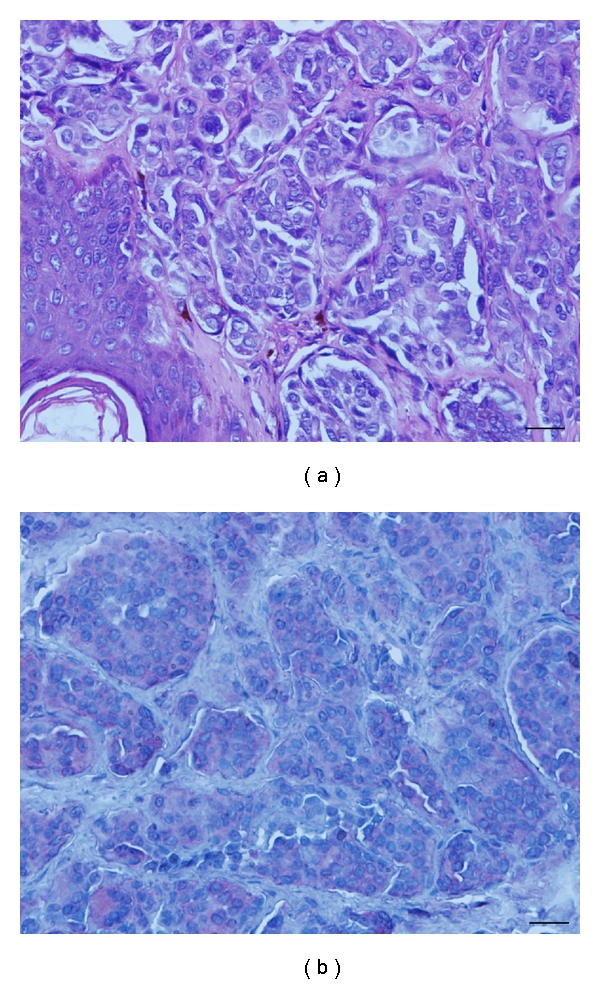
Hematoxylin and eosin staining of benign melanocytic nevi (a). Immunorecognition of 14F7 Mab (b). Note that a weak to moderate (finely granular) reactivity located on plasmatic membrane and cytoplasm of nevi cells. Black bar = 100 *μ*m.

The 14F7 immunostaining was observed in 2/11 (18.2%) of benign melanocytic nevi. A weak to moderate reaction following a homogeneous and finely granular pattern in 2/6 (33.3%) of compound nevi was detected. This recognition was located in the plasmatic membrane and also in the cytoplasm of more than 50% of melanocytes (Figure 1). No immunoreaction in melanocytes from others benign nevi such as intradermic (0/4), and junctional (0/1) was observed. 

A weak immunorecognition of 14F7 Mab in 1/7 (14.3%) of dysplastic melanocytic nevi was evidenced. The staining was located mainly in the plasmatic membrane and also in the cytoplasm of melanocytes (data no shown). When the number of positive samples, the intensity range of reaction, and the percentages of positive cells in BMN versus DMN were compared, no statistically significant differences were observed (*P* = .1851 by Fisher's Exact Test, *P* = .4150, and *P* = .4731 by chi-square Test, resp.).

### 3.4. Immunohistochemical Staining in Some Malignant Tissues from Human Skin

Table 2 shows the results of 14F7 Mab immunoreaction in some malignancies derived from human skin.

### 3.5. Basal Cell Carcinoma

**Figure 2:**
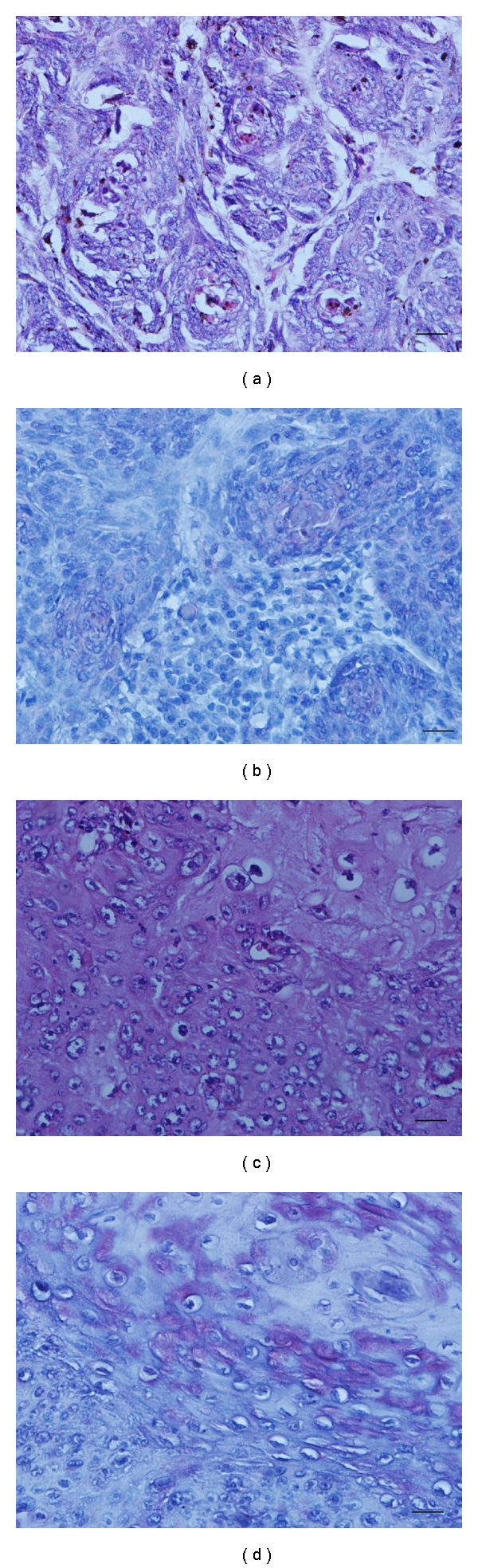
Hematoxylin and eosin staining of basal cell carcinoma (a) and squamous cell carcinoma of human skin (c). A moderate immunostaining with 14F7 Mab was detected on basal cell carcinoma mostly located in the center of the typical lobules and columns of malignant basaloid cells (b). The squamous cell carcinoma showed an intense reaction becoming into more intense near the central keratinization and horn pearl formation (d). Black bar =100 *μ*m.

A weak to moderate staining with 14F7 Mab in 2/13 (15.4%) of basal cell carcinoma was observed. This recognition was evidenced homogeneous and finely granular and was located mainly in the plasmatic membrane and also in the cytoplasm of malignant keratinocytes. This pattern was described mostly in the center of the typical lobules and columns of malignant basaloid cells (Figure 2).

### 3.6. Squamous Cell Carcinoma

A weak to intense immunostaining in more than 5% of tumoral cells in 4/8 (50%) of squamous cell carcinomas was evidenced. The immunorecognition of 14F7 was homogeneous and finely granular and was located in the membrane and the cytoplasm of malignant keratinocytes. Two of these samples showed a weak reaction in 5% of tumor cells, while an intense staining was observed in 30–40% of these malignant cells from the rest of the positive tumors. The reactivity of 14F7 Mab became more intense near the central keratinization and horn pearl formation (Figure 2) in the last two samples. 

No statistically significant differences in the 14F7 reaction was detected when the number of positive samples in BCC versus SCC were compared (*P* = .1006 by Fisher's Exact Test), neither when the intensity range of reaction and the percentages of positive cells were analyzed (*P* = .0920 and *P* = .3062 by chi-square Test, resp.).

### 3.7. Cutaneous Malignant Melanoma

**Figure 3:**
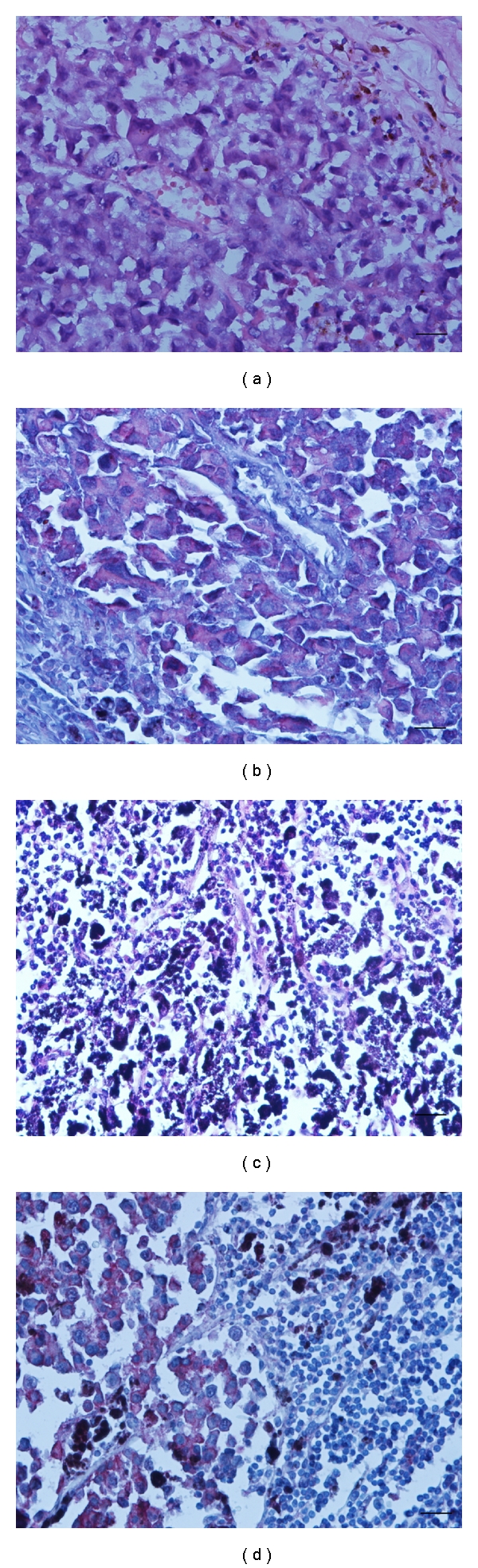
Hematoxylin and eosin staining of cutaneous malignant melanoma (a) and lymph node metastase (c). See a strong and finely granular immunoreactivity of 14F7 Mab in cutaneous malignant melanoma (b) as well as, in lymph node metastases (d). The reaction was located on plasmatic membrane and cytoplasm. Black bar =100 *μ*m.

An intense homogeneous and finely granular pattern of recognition was present in all CMM tested (28/28), 27 of which were melanotic and 1, amelanotic (Figure 3). The reaction was located mainly on the plasmatic membrane in more than 50% of malignant melanocytes, although the cytoplasm of these cells was also stained. 

The immunoreactivity of 14F7 Mab show a statistically significant differences when BMN + DMN versus CMM and when BCC + SCC versus CMM were compared in function of all parameters measured (*P* = .0000 by Fisher's Exact Test and by chi-square Test).

### 3.8. Lymph Node Metastases

A moderate to intense reactivity of 14F7 Mab was detected in more than 50% of tumoral cells in 5/7 (71.4%) of lymph node metastases. The recognition of 14F7 was homogeneous and finely granular and was located in the membrane and the cytoplasm of malignant melanocytes (Figure 3). A heterogeneous pattern was evidenced in one sample, exhibiting a moderate reaction with 14F7 in 6–25% of malignant cells. 

No statistically significant different in CMM versus LNM was evidenced when the number of positive sample was analyzed (*P* = .2059 by Fisher's Exact Test). On the contrary, when the intensity range of reaction and the percentages of positive cells were compared, statistically significant differences were detected (*P* = .0002 and *P* = .0166 by chi-square Test, resp.).

## 4. Discussion

Gangliosides are glycosphingolipids containing sialic acid, widely distributed in human tissues of both normal and tumoral neuroectodermal origin [4, 16]. These molecules have been considered attractive targets for cancer immunotherapy and diagnosis based on their higher expression in tumors as compared with normal tissues, as well as, their relevance in tumoral growth [9]. 

It is known that GM3, GM2, and GD3 are the predominant gangliosides in most of the samples of normal keratinocytes, although immunohistochemical studies using anti-GM3 (M2590) and anti-GD3 (R24) monoclonal antibodies showed a just weak recognition of these cells. Similar results were obtained in squamous cell carcinoma and basal cell carcinoma of human skin [17]. Additionally, the expression of 9-O-Ac-GD3 ganglioside, not expressed in normal keratinocytes, was detected in basal cell carcinoma [18]. On the other hand, the expression of little amounts of N-glycolyl neuraminic acid in this malignant neoplasm has been also reported [19]. In addition, gangliosides are shed in substantial quantities into the tumor microenvironment. Afterward, they are taken up and bnd efficiently to host cells that are found in this microenvironment [20]. 

N-glycolyl neuraminic acid has been considered to be absent in normal human tissues [21] due to a partial deletion of the gene coding for the production of CMP-Neu5Ac hydroxylase [22], although the expression of N-glycolyl gangliosides has been reported in various malignancies [8, 10, 11, 23]. The expression of N-glycolyl neuraminic acid residues in neoplastic cells has been associated with its incorporation, by mean of dietary sources, to the accelerated metabolism of these cells [24, 25]. Moreover, some authors have suggested an alternative pathway to the Neu5Gc synthesis from others intermediates of cellular metabolism in some human tumors [26].

In our study, we confirmed the lack of reactivity of 14F7 Mab in normal skin samples as we previously published [10]. This result is consistent with the absence of N-glycolyl function in normal keratinocytes [27]. It is known that GM3 ganglioside is a normal component of plasmatic membrane of normal human cells [28], including keratinocytes [17, 29]. Therefore, we also confirmed that 14F7 is able to distinguish between the N-glycolyl and the N-acetyl functions of the GM3 ganglioside [10]. 

BCC and SCC are malignant neoplasms arising from the keratinocytes, cells of ectodermal origin that usually show a slower and less invasive cellular growth than CMM [2]. Nevertheless, it is known that some SCC can be biologically aggressive, showing a greater propensity for local recurrence and metastatic potential [30], while most of BCC are less aggressive than SSC and their prognosis is very good [2]. Here we described a low recognition of 14F7 in BCC and SCC. These results are in agreement with the lack of recognition of P3 Mab in BCC and SCC (a murine Mab specific for N-glycolylneuraminic acid-containing gangliosides that also recognizes sulfated glycolipids) [11], as well as with previous reports of Carr et al. in epidermoid carcinoma using 14F7 MAb [10].

The tissue oxygen content changes gradually with distance from blood vessels as occurs in SCC [31]. The influence of tumor hypoxia in the formation, progression and evolution of cutaneous melanoma to a more aggressive form of the disease has been previously reported [31]. Tumor hypoxia induces the expression of NeuGcGM2 gangliosides in human cancer cells through the incorporation of the nonhuman sialic acid NeuGc, supporting the idea that the specific effect of hypoxia is to expedite sialic acid transport from the external medium, in relation with the increment of sialin expression (a sialic acid transport) [32]. The 14F7 immunoreactivity mainly located in the center of the typical lobules and columns of malignant basaloid cells and close to the central keratinization and horn pearl formation in two of SCC could suggest a higher expression of NeuGc function in these areas as well as a possible relationship with tumor hypoxia. 

It is well known that melanoma is a malignant neoplasm arising from the melanocytes, cells of neuro-ectodermal origin, which show a very aggressive biological behavior and poor prognosis [3]. Numerous efforts have been done to demonstrate the relationship between melanocytic nevi and malignant melanoma. In addition, some immunohistochemical methods have been used to distinguish between normal skin, melanocytic nevi, and primary melanoma, based in some changes in expression and/or composition of gangliosides [16, 33–35]. 

Some author have reported aberrant expression of gangliosidos in melanocytes neoplasias [16, 33, 35, 36]. Although the major gangliosides expression studies in human malignancies have been restricted to N-acetylated variant of sialic acid [33, 34, 36]. Hanganutziu-Deicher (HD) antigens have been reported to be absent in normal human tissues and melanocytic nevus, but can be expressed on a variety of human malignant cells, including melanoma [27, 37, 38]. HD antigen is classified as a heterophile antigen and chemically defined as a ganglioside and/or glycoprotein (glycoconjugates) which contains N-glycolylneuraminic acid (NeuGc) [37]. Here we obtained a low immunostaining in BMN as well as in DMN. 

All CMM studied and lymph node metastases reacted intensely with the 14F7. The immunostaining pattern was similar to previous reports from our group [10, 12]. However, Kawachi and Saida suggested that HD antigen is expressed on the carbohydrate chains of glycoproteins but not on those of gangliosides in human melanoma [39]. It is known glycolipids are partially or completely extracted from the tissues after ethanol and absolute methanol treatment. Additionally, gangliosides are lowly expressed in human skin and they represent only the 0.1 percent of the epidermal lipids [40]. These results suggest that probably 14F7 Mab cross-reacts with other glycoconjugate containing NeuGc. 

In summary, we reported an intense recognition of 14F7 Mab in cutaneous melanomas and lymph nodes metastases, while, the rest of the entities showed a limited reaction. These results suggest that 14F7 reactivity could be closely related with the more aggressive biological behavior of melanocytes-derived tumors. Experiments looking for a better understanding of the 14F7 recognition and its relationship with the biological behavior of these malignant tumors, as well as, for the evaluation of the chemical nature of the antigenic determinant recognized by 14F7 Mab are ongoing. In addition, clinical trials with NeuGcGM3/VSSP molecular cancer vaccine in melanoma patients are ongoing in our country.

## 5. Conclusions

The recognition of 14F7 Mab in cutaneous malignant melanoma and lymph node metastases as well as its limited reaction in normal sections and other entities of human skin suggests a possible relationship between the 14F7 reactivity with the more aggressive behavior of malignant tumor of melanocytes. Our data could support the possible use of NeuGcGM3 as target for both active and passive immunotherapy of malignant melanoma expressing this molecule.

## Figures and Tables

**Table 1:** Immunorecognition of 14F7 Mab in normal and non-malignant lesions of human skin.

Samples	No. cases (%)	Range intensity	Positive cells
(i) Normal skin	0/9 (0)	−	0
(ii) Nevi			
Benign melanocytic nevi	2/11 (18,2)	+/++	3
Dermal	0/4 (0)	−	0
Junctional	0/1 (0)	−	0
Compound	2/6 (33,3)	+/++	3
Displastic melanocytic nevi	1/7 (14,3)	+	1

Intensity: − negative, + weak, ++ moderate, +++ intense. Positive cells: 0 (negative to less than 5%), 1 (6–25%), 2 (26–50%) and 3 (more than 50%).

**Table 2:** Immunorecognition of 14F7 Mab in malignant lesions derived from human skin.

Samples	No. cases (%)	Range intensity	Positive cells
(i) Nonmelanoma skin tumors			
Basal cell carcinoma	2/13 (15.4)	+/++	1
Solid	0/7	−	0
Adenoid	2/5 (40)	+/++	1
Keratotic	0/1	−	0
Squamous cell carcinoma	4/8 (50)	+/+++	1/2
Well differentiated	2/6 (33.3)	+++	2
Ulcerated	2/2	+	1
(ii) Cutaneous malignant melanoma	28/28 (100)	+++	3
Melanotic	27/27	+++	3
Amelanotic	1/1	+++	3
(iii) Lymph node metastases	6/7 (85.7)	++/+++	1/3

Intensity: − negative, + weak, ++ moderate, +++ intense. Positive cells: 0 (negative to less than 5%), 1 (6–25%), 2 (26–50%) and 3 (more than 50%).

## References

[1] Jemal A, Siegel R, Ward E, Murray T, Xu J, Thun MJ (2007). Cancer statistics, 2007. *Ca: A Cancer Journal for Clinicians*.

[2] Martinez MAR, Francisco G, Cabral LS, Ruiz IRG, Neto CF (2006). Molecular genetics of non-melanoma skin cancerGenética molecular aplicada ao câncer cutâneo não melanoma. *Anais Brasileiros de Dermatologia*.

[3] Balch CM, Gershenwald JE, Soong SJ (2009). Final version of 2009 AJCC melanoma staging and classification. *Journal of Clinical Oncology*.

[4] Yamashita T, Wada R, Sasaki T (1999). A vital role for glycosphingolipid synthesis during development and differentiation. *Proceedings of the National Academy of Sciences of the United States of America*.

[5] Birklé S, Zeng G, Gao L, Yu RK, Aubry J (2003). Role of tumor-associated gangliosides in cancer progression. *Biochimie*.

[6] Higashi H, Sasabe T, Fukui Y, Maru M, Kato S (1988). Detection of gangliosides as N-glycolylneuraminic acid-specific tumor-associated Hanganutziu-Deicher antigen in human retinoblastoma cells. *Japanese Journal of Cancer Research*.

[7] Miyake M, Hashimoto K, Ito M (1990). The abnormal occurrence and the differentiation-dependent distribution of N-acetyl and N-glycolyl species of the ganglioside GM2 in human germ cell tumors. A study with specific monoclonal antibodies. *Cancer*.

[8] Watarai S, Kushi Y, Shigeto R (1995). Production of monoclonal antibodies directed to Hanganutziu-Deicher active gangliosides, N-glycolylneuraminic acid-containing gangliosides. *Journal of Biochemistry*.

[9] Zhang S, Cordon Cardo C, Zhang HS (1997). Selection of carbohydrate tumour antigens as targets for immune attack using immunohistochemistry. I. Focus on gangliosides. *International Journal of Cancer*.

[10] Carr A, Mullet A, Mazorra Z (2000). A mouse IgG monoclonal antibody specific for N-glycolyl GM3 ganglioside recognized breast and melanoma tumors. *Hybridoma*.

[11] Vazquez AM, Alfonso M, Lanne B (1995). Generation of a murine monoclonal antibody
specific for N-glycolylneuraminic acid-containing gangliosides that also recognizes sulfated glycolipids. *Hybridoma*.

[12] Osorio M, Gracia E, Rodríguez E (2008). Heterophilic NeuGcGM3 ganglioside cancer vaccine in advanced melanoma patients: results of a phase Ib/IIa study. *Cancer Biology and Therapy*.

[13] Blanco R, Rengifo E, Cedeno M, Rengifo ChE, Alonso DF, Carr A (2011). Immunoreactivity of the 14F7 Mab raised against N-glycolyl GM3 ganglioside in epithelial malignant tumors from digestive system. *ISRN Gastroenterology*.

[14] van Cruijsen H, Ruiz M, van der Valk P, de Gruijl TD, Giaccone G (2009). Tissue micro array analysis of ganglioside *N*-glycolyl GM3 expression and signal transducer and activator of transcription (STAT)-3 activation in relation to dendritic cell infiltration and microvessel density in non-small cell lung cancer. *BMC Cancer*.

[15] Scursoni AM, Galluzzo L, Camarero S (2010). Detection and characterization of N-glycolyated gangliosides in Wilms tumor by immunohistochemistry. *Pediatric and Developmental Pathology*.

[16] Kohla G, Stockfleth E, Schauer R (2002). Gangliosides with O-acetylated sialic acids in tumors of neuroectodermal origin. *Neurochemical Research*.

[17] Paller AS, Arnsmeier SL, Robinson JK, Bremer EG (1992). Alteration in keratinocyte ganglioside content in basal cell carcinomas. *Journal of Investigative Dermatology*.

[18] Heidenheim M, Hansen ER, Baadsgaard O (1995). CDw60, which identifies the acetylated form of GD3 gangliosides, is strongly expressed in human basal cell carcinoma. *British Journal of Dermatology*.

[19] Fahr C, Schauer R (2001). Detection of sialic acids and gangliosides with special reference to 9-*O*-acetylated species in basaliomas and normal human skin. *Journal of Investigative Dermatology*.

[20] Liu Y, McCarthy J, Ladisch S (2006). Membrane ganglioside enrichment lowers the threshold for vascular endothelial cell angiogenic signaling. *Cancer Research*.

[21] Kawai T, Kato A, Higashi H, Kato S, Naiki M (1991). Quantitative determination of N-glycolylneuraminic acid expression in human cancerous tissues and avian lymphoma cell lines as a tumor-associated sialic acid by gas chromatography-mass spectrometry. *Cancer Research*.

[22] Irie A, Koyamat S, Kozutsumi Y, Kawasaki T, Suzuki A (1998). The molecular basis for the absence of N-glycolylneuraminic acid in humans. *Journal of Biological Chemistry*.

[23] Marquina G, Waki H, Fernandez LE (1996). Gangliosides expressed in human breast cancer. *Cancer Research*.

[24] Tangvoranuntakul P, Gagneux P, Diaz S (2003). Human uptake and incorporation of an immunogenic nonhuman dietary sialic acid. *Proceedings of the National Academy of Sciences of the United States of America*.

[25] Bardor M, Nguyen DH, Diaz S, Varki A (2005). Mechanism of uptake and incorporation of the non-human sialic acid N-glycolylneuraminic acid into human cells. *Journal of Biological Chemistry*.

[26] Malykh YN, Schauer R, Shaw L (2001). N-glycolylneuraminic acid in human tumours. *Biochimie*.

[27] Saida T, Ikegawa S, Takizawa Y, Kawachi S (1990). Immunohistochemical detection of heterophile Hanganutziu-Deicher antigen in human malignant melanoma. *Archives of Dermatological Research*.

[28] Higashi H, Naiki M, Matuo S, Okouchi K (1977). Antigen of ‘serum sickness’ type of heterophile antibodies in human sera: identification as gangliosides with N-glycolylneuraminic acid. *Biochemical and Biophysical Research Communications*.

[29] Nakakuma H, Horikawa K, Kawaguchi T (1992). Common phenotypic expression of gangliosides GM3 and GD3 in normal human tissues and neoplastic skin lesions. *Japanese Journal of Clinical Oncology*.

[30] Mullen JT, Feng L, Xing Y (2006). Invasive squamous cell carcinoma of the skin: defining a high-risk group. *Annals of Surgical Oncology*.

[31] Evans SM, Hahn S, Pook DR (2000). Detection of hypoxia in human squamous cell carcinoma by EF5 binding. *Cancer Research*.

[32] Yin J, Hashimoto A, Izawa M (2006). Hypoxic culture induces expression of sialin, a sialic acid transporter, and cancer-associated gangliosides containing non-human sialic acid on human cancer cells. *Cancer Research*.

[33] Carubia JM, Yu RK, Macala LJ (1984). Gangliosides of normal and neoplastic human melanocytes. *Biochemical and Biophysical Research Communications*.

[34] Cheresh DA, Reisfeld RA, Varki AP (1984). O-acetylation of disialoganglioside GD3 by human melanoma cells creates a unique antigenic determinant. *Science*.

[35] Dippold WG, Dienes HP, Knuth A (1985). Immunohistochemical localization of ganglioside GD3 in human malignant melanoma, epithelial tumors, and normal tissues. *Cancer Research*.

[36] Tsuchida T, Saxton RE, Irie RF (1987). Gangliosides of human melanoma: GM2 and tumorigenicity. *Journal of the National Cancer Institute*.

[37] Nakarai H, Chandler PJ, Kano K, Morton DL, Irie RF (1990). Hanganutziu-Deicher antigen as a possible target for immunotherapy of melanoma. *International Archives of Allergy and Applied Immunology*.

[38] Alfonso M, Díaz A, Hernández AM (2002). An anti-idiotype vaccine elicits a specific response to N-glycolyl sialic acid residues of glycoconjugates in melanoma patients. *Journal of Immunology*.

[39] Kawachi S, Saida T (1992). Analysis of the expression of Hanganutziu-Deicher (HD) antigen in human malignant melanoma. *Journal of Dermatology*.

[40] Boddé HE, Holman B, Spies F (1990). Freeze-fracture electron microscopy of in vitro reconstructed human epidermis. *Journal of Investigative Dermatology*.

